# The 2026 ACC/AHA Dyslipidemia Guideline: A Critical and Transatlantic Perspective on Personalized Lipid Management

**DOI:** 10.3390/life16091470

**Published:** 2026-09-03

**Authors:** Pierre Sabouret, Dario Mafrica, Domenico Mario Giamundo, Joanna Popiolek Kalisz, Elad Asher, Dan Atar, Giuseppe Ando

**Affiliations:** 1 Heart Institute and Action Group, Pitié-Salpêtrière Hospital, Sorbonne University, 75013 Paris, France; 2 Department of Clinical, Internal, Anesthesiological and Cardiovascular Sciences, Sapienza University of Rome, 00185 Rome, Italy; dariomafrica@gmail.com; 3 Division of Cardiology, Policlinico Casilino, 00169 Rome, Italy; jamundus20@libero.it; 4 Clinical Dietetics Unit, Department of Bioanalytics, Medical University of Lublin, Department of Cardiology, Cardinal Wyszynski Hospital in Lublin, ul. Chodzki 7, 20-059 Lublin, Poland; joanna.popiolek-kalisz@umlub.edu.pl; 5 Jesselson Integrated Heart Center, The Eisenberg R&D Authority, Shaare Zedek Medical Center and Faculty of Medicine, Hebrew University of Jerusalem, Jerusalem 9103102, Israel; easher@szmc.org.il; 6 Department of Cardiology, Oslo University Hospital Ullevål, Kirkeveien 166, 0450 Oslo, Norway; dan.atar@medisin.uio.no; 7 Institute of Clinical Medicine, University of Oslo, P.O. Box 1171 Blindern, 0318 Oslo, Norway; 8 Department of Clinical and Experimental Medicine, University of Messina, AOU Policlinico “Gaetano Martino”, 98125 Messina, Italy; giuseppe.ando@unime.it

**Keywords:** dyslipidemia, LDL cholesterol, PREVENT-ASCVD, lipoprotein(a), coronary artery calcium, lipid-lowering therapy

## Abstract

The 2026 ACC/AHA Multisociety Guideline marks a major shift in dyslipidemia management, extending the traditional LDL-C-centered approach to include non-HDL-C, ApoB, lipoprotein(a), triglyceride-rich lipoproteins, inherited lipid disorders, and severe hypertriglyceridemia. The introduction of PREVENT-ASCVD, the renewed use of explicit lipid targets, and the structured integration of risk enhancers and coronary artery calcium support a more individualized approach to cardiovascular prevention. This review critically examines the guideline’s main innovations, with particular attention to the distinction between recommendations supported by cardiovascular outcome evidence and those relying mainly on observational data, surrogate endpoints, or extrapolation. It also compares the US framework with the 2025 ESC/EAS Focused Update, highlighting growing agreement on LDL-C goals but persistent differences in risk estimation, imaging, biomarkers, and treatment pathways. Finally, we discuss unresolved issues involving implementation, adherence, access, cost, health equity, inclisiran outcomes, and emerging lipoprotein(a)-lowering therapies. The guideline’s impact will depend on translating greater precision into timely, feasible, and equitable care.

## 1. Introduction

The 2026 ACC/AHA Multisociety Guideline on the Management of Dyslipidemia represents the first comprehensive revision of US lipid recommendations since the 2018 guideline on blood cholesterol. Its broader scope extends beyond LDL cholesterol (LDL-C) and statin-based treatment decisions to include non-HDL-C, triglyceride-rich lipoproteins, apolipoprotein B (ApoB), lipoprotein(a) [Lp(a)], inherited lipid disorders, and severe hypertriglyceridemia. This broader perspective reflects the recognition that LDL-C alone may not fully capture atherogenic risk, particularly in patients with diabetes, obesity, chronic kidney disease, mixed dyslipidemia, or elevated Lp(a) [1,2]. A central principle of the new guideline is that cardiovascular risk depends on both the concentration and duration of exposure to ApoB-containing lipoproteins. Evidence from genetic studies, epidemiological cohorts, meta-analyses, and randomized trials supports a causal and cumulative relationship between atherogenic lipoproteins and atherosclerotic cardiovascular disease (ASCVD). These findings provide the rationale for earlier intervention and sustained lipid control rather than postponing treatment until short-term risk becomes high [3,4,5,6,7]. Another major change is the replacement of the Pooled Cohort Equations with PREVENT-ASCVD in primary prevention. PREVENT estimates both 10-year and 30-year risk in adults aged 30–79 years and incorporates contemporary cardiovascular, renal, and metabolic variables through sex-specific, race-free equations. The guideline then refines this estimate through risk-enhancing factors and, when clinically appropriate, selective measurement of Lp(a), ApoB, and coronary artery calcium [1,8]. The return to explicit LDL-C and non-HDL-C goals also brings the US recommendations closer to the target-based European framework. Nevertheless, relevant differences remain in risk classification, the use of imaging and biomarkers, and the timing and sequencing of combination therapy. These differences warrant direct comparison with the 2025 ESC/EAS Focused Update [1,9]. This review critically examines the main innovations of the 2026 ACC/AHA guideline, including long-term risk assessment, lipid targets, treatment selection, special populations, and implementation. It also explores areas of convergence and divergence with contemporary European recommendations and discusses whether greater diagnostic and therapeutic precision can be translated into timely, feasible, and equitable cardiovascular prevention.

Rather than reproducing the recommendations in a guideline-at-a-glance format, we use the 2026 document as a starting point to examine how strongly its main changes are supported by outcome data and where uncertainty remains. Particular attention is given to differences with the European approach, to recommendations based largely on observational evidence or surrogate lipid endpoints, and to the practical consequences of adding further biomarkers, imaging tests, and treatment options to routine care. This perspective is intended to complement the primary guideline documents by linking recommendations to the strength of the underlying evidence and to the barriers that may limit their implementation.

### Literature Search and Evidence Selection

This narrative review was informed by a targeted search of PubMed/MEDLINE and the reference lists of the 2026 ACC/AHA and 2025 ESC/EAS documents. The literature search covered publications available through August 2026, with no predefined lower date limit. Search terms included combinations of dyslipidemia, LDL-C, non-HDL-C, ApoB, lipoprotein(a), PREVENT, coronary artery calcium, PCSK9, inclisiran, bempedoic acid, hypertriglyceridemia, and cardiovascular outcomes. Priority was given to current guidelines, randomized cardiovascular outcome trials, major meta-analyses, large validation or observational cohorts, and studies directly informing new or revised recommendations. Earlier landmark studies were retained when necessary to establish the evidence base for existing treatment principles. ClinicalTrials.gov was also checked for the current status of ongoing outcome trials. Because this was a narrative review, study selection was not based on a formal systematic-review protocol.

## 2. Risk Assessment in Primary Prevention: PREVENT-ASCVD, CAC, and Lipid Biomarkers

### 2.1. PREVENT-ASCVD: Contemporary Short- and Long-Term Risk Estimation

The 2026 ACC/AHA guideline replaces the Pooled Cohort Equations with PREVENT-ASCVD for adults aged 30–79 years without known cardiovascular disease. PREVENT provides 10-year and, when applicable, 30-year risk estimates through contemporary, sex-specific, race-free models. Alongside traditional risk factors, the base equation incorporates body mass index, estimated glomerular filtration rate, and current lipid-lowering and antihypertensive treatment; urinary albumin-to-creatinine ratio, glycated hemoglobin, and the Social Deprivation Index may further refine risk when available. Ten-year risk is classified as low (<3%), borderline (3% to <5%), intermediate (5% to <10%), or high (≥10%) [1,8].

PREVENT should not be interpreted as a lifetime-risk calculator or as a stand-alone treatment rule. Its main advantage is the combination of short- and longer-term risk estimation, which may identify younger adults with low 10-year risk but substantial cumulative exposure to atherogenic lipoproteins. In borderline- or intermediate-risk individuals, the estimate should be integrated with risk-enhancing factors, including premature family history of ASCVD, persistent LDL-C elevation, chronic inflammatory disease, CKD, adverse pregnancy-related conditions, and selected lipid biomarkers. Although its race-free design avoids treating race as a biological variable, it does not eliminate disparities in access to preventive care. Recent multinational validation showed generally favorable performance across several regions, but representation remained limited in some populations and local recalibration may still be required before US thresholds are adopted in other healthcare systems [8,10].

### 2.2. Coronary Artery Calcium as a Tool for Risk Reclassification

CAC has a more prominent role when the decision to initiate or intensify lipid-lowering therapy remains uncertain after clinical assessment. It should therefore be used selectively rather than as a population-wide screening test. CAC provides a direct measure of calcified coronary atherosclerotic burden and adds prognostic information beyond conventional risk factors. A CAC score of zero may support temporary treatment deferral in selected patients without major high-risk conditions, whereas detectable CAC increasingly favours pharmacological treatment as the burden rises [1,11].

CAC ≥ 1000 Agatston units identifies an extreme-risk phenotype with event rates approaching those observed in some stable secondary-prevention populations. The guideline supports an LDL-C reduction of at least 50% and a goal below 55 mg/dL in this setting [1,12]. However, these recommendations rely mainly on observational risk estimates and extrapolation from outcome trials rather than randomized comparisons of CAC-guided targets. Cost, radiation exposure, incidental findings, availability, and potential inequities should therefore be considered before testing.

### 2.3. Lipoprotein(a): From Risk Enhancer to Universal Adult Screening

Measurement of Lp(a) at least once in adulthood is among the most clinically relevant changes. Lp(a) is largely genetically determined, remains relatively stable throughout life, and is causally associated with ASCVD and calcific aortic valve disease. An Lp(a) concentration ≥ 125 nmol/L or ≥50 mg/dL is considered a risk-enhancing factor, although the two units are not directly interchangeable because the relationship between mass and particle concentration varies with apolipoprotein(a) isoform size. Risk increases continuously across the Lp(a) distribution, so markedly elevated values should carry greater clinical weight than concentrations just above the conventional threshold [1,13].

Lp(a) and CAC provide complementary information: the former reflects inherited lifelong exposure, whereas the latter demonstrates established coronary atherosclerotic burden. Their coexistence should not automatically be labelled a formally defined very-high-risk category. Until dedicated outcome trials establish the benefit of specific Lp(a)-lowering therapies, elevated Lp(a) should prompt earlier and more intensive control of LDL-C and all other modifiable risk factors [1].

### 2.4. Apolipoprotein B and Residual Atherogenic Risk

ApoB estimates the total number of circulating atherogenic particles because each LDL, very-low-density lipoprotein remnant, intermediate-density lipoprotein, and Lp(a) particle carries one ApoB molecule. LDL-C measures the cholesterol content of LDL particles and may therefore underestimate particle burden when numerous cholesterol-depleted particles are present. This discordance is particularly relevant in hypertriglyceridemia, type 2 diabetes, obesity, cardiovascular–kidney–metabolic syndrome, and treated ASCVD. In statin-treated patients, residual risk may align more closely with ApoB and non-HDL-C than with LDL-C when these measures are discordant [14].

**Figure 1:**
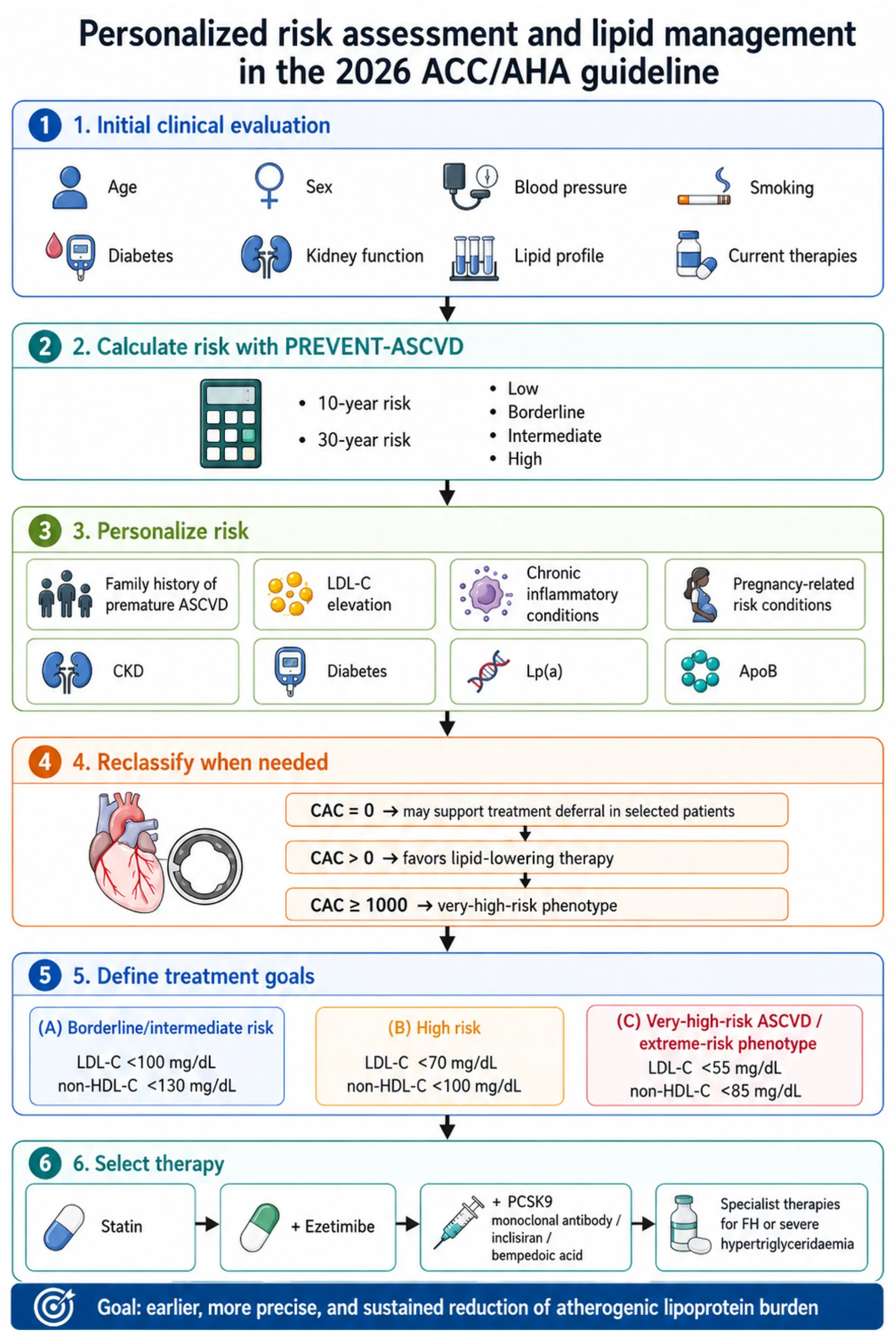
**Personalized risk assessment and lipid management in the 2026 ACC/AHA guideline**. The 2026 ACC/AHA framework for dyslipidemia management moves from a single-score approach to a layered strategy integrating initial clinical evaluation, PREVENT-ASCVD risk estimation, risk-enhancing factors, selective use of lipoprotein(a) [Lp(a)] and apolipoprotein B (ApoB), and coronary artery calcium (CAC) for risk reclassification. The resulting risk profile informs the definition of LDL-C and non-HDL-C treatment goals and the selection of lipid-lowering therapy, from statins to combination treatment and specialist therapies in selected phenotypes. Overall, this model supports earlier, more precise, and sustained reduction in atherogenic lipoprotein burden. **Abbreviations:** ACC, American College of Cardiology; AHA, American Heart Association; ApoB, apolipoprotein B; ASCVD, atherosclerotic cardiovascular disease; CAC, coronary artery calcium; CKD, chronic kidney disease; LDL-C, low-density lipoprotein cholesterol; Lp(a), lipoprotein(a); PREVENT-ASCVD, Predicting Risk of cardiovascular disease EVENTs–Atherosclerotic Cardiovascular Disease.

The guideline does not recommend universal ApoB testing. Selective measurement is the most useful when LDL-C may incompletely represent atherogenic burden or when residual risk persists despite achievement of conventional lipid goals. The overall strategy therefore moves from a single-score model to layered assessment with PREVENT-ASCVD, risk enhancers, CAC, Lp(a), and selective ApoB testing. The ACC/AHA pathway from risk estimation to treatment selection is illustrated in Figure 1. Its value will depend on using additional tests only when they are likely to change management and on linking abnormal findings to clear, accessible treatment pathways.

The layered approach is clinically appealing, but its incremental value is unlikely to be uniform across patients. PREVENT, Lp(a), ApoB, and CAC answer different clinical questions and should not become a checklist applied irrespective of the degree of initial uncertainty. Their prognostic value is better established than the ability of a strategy combining several of these tools to improve cardiovascular outcomes or cost-effectiveness. Additional testing is therefore most useful when the result is likely to change the decision to initiate or intensify treatment; otherwise, greater diagnostic precision may add complexity and cost without materially changing management.

## 3. LDL-C and Non-HDL-C Goals: The Return of a Target-Based Framework

### 3.1. Risk-Based LDL-C and Non-HDL-C Goals

The reintroduction of explicit LDL-C and non-HDL-C goals is one of the most visible changes in the 2026 ACC/AHA guideline. The 2013 and 2018 US recommendations emphasized statin intensity, expected percentage reduction, and LDL-C thresholds for adding non-statin therapy rather than a conventional treat-to-target strategy. The new document retains these elements but combines them with absolute lipid goals tailored to cardiovascular risk [1,2]. In primary prevention, adults at borderline or intermediate 10-year risk should generally achieve LDL-C below 100 mg/dL and non-HDL-C below 130 mg/dL. For those at high risk, the corresponding goals are below 70 mg/dL and below 100 mg/dL. A 30–49% LDL-C reduction is generally appropriate in borderline or intermediate risk, whereas a reduction of at least 50% is expected in high-risk patients [1]. In secondary prevention, patients at very high risk should achieve LDL-C below 55 mg/dL and non-HDL-C below 85 mg/dL. An LDL-C goal below 70 mg/dL remains appropriate for patients with established ASCVD who do not meet very-high-risk criteria. Because many patients with previous cardiovascular events have additional high-risk features, a substantial proportion of the secondary-prevention population will qualify for the more intensive target [1]. Absolute goals provide a measurable therapeutic objective, facilitate assessment of treatment response, and identify patients who require intensification. Percentage reduction remains complementary because the same achieved LDL-C level may reflect markedly different responses according to the untreated baseline concentration. Therapeutic adequacy should therefore consider baseline LDL-C, percentage reduction, achieved lipid levels, and overall cardiovascular risk.

### 3.2. Evidence Supporting Intensive LDL-C Lowering

Most evidence supporting intensive LDL-C lowering comes from trials comparing therapeutic strategies rather than assigning patients to fixed lipid targets. IMPROVE-IT showed that adding ezetimibe to simvastatin after acute coronary syndrome produced further LDL-C reduction and a modest but significant decrease in cardiovascular events, whereas FOURIER and ODYSSEY OUTCOMES demonstrated additional cardiovascular benefit with PCSK9 inhibition in patients with stable ASCVD and after recent acute coronary syndrome, respectively [6,15,16]. Evidence from atherosclerotic cerebrovascular disease also supports a target-based approach. Treat Stroke to Target showed that, among patients with recent ischemic stroke or transient ischemic attack and documented atherosclerosis, an LDL-C target below 70 mg/dL was superior to a target of 90–110 mg/dL in preventing subsequent major cardiovascular events [17]. Benefits also appear to continue when LDL-C is reduced from already low concentrations. A meta-analysis of statin and non-statin outcome trials found a consistent reduction in major vascular events among populations starting with median LDL-C levels of 70 mg/dL or lower, without a corresponding increase in major safety outcomes during the available follow-up [18]. FOURIER-OLE further supports the long-term efficacy and overall safety of sustained exposure to very low LDL-C levels [7]. These findings support progressively lower ASCVD risk with greater LDL-C reduction, but the absolute benefit is not identical for every patient. It depends on baseline cardiovascular risk, the magnitude and duration of LDL-C reduction, adherence, competing risks, and life expectancy.

### 3.3. The Complementary Role of Non-HDL-C

Non-HDL-C reflects the cholesterol carried by all ApoB-containing atherogenic particles, including LDL, very-low-density lipoprotein remnants, intermediate-density lipoproteins, and Lp(a). It is particularly useful in patients with hypertriglyceridemia, type 2 diabetes, obesity, metabolic syndrome, or mixed dyslipidemia, in whom LDL-C may underestimate the residual atherogenic burden. The non-HDL-C goal is set 30 mg/dL above the corresponding LDL-C target across the principal risk categories [1]. Non-HDL-C and ApoB are related but not interchangeable. Non-HDL-C measures the cholesterol content of atherogenic particles, whereas ApoB estimates their number. Selective ApoB measurement therefore remains useful when LDL-C and non-HDL-C appear discordant with the patient’s metabolic phenotype. The return to explicit targets improves therapeutic clarity, but it should not be interpreted as evidence that every numerical threshold has been independently validated in randomized trials. With the exception of selected target-based studies such as Treat Stroke to Target, most evidence derives from trials of specific lipid-lowering therapies and from the consistent relationship between the magnitude of LDL-C reduction and cardiovascular risk reduction. Lipid goals should therefore be viewed as practical treatment objectives derived from the totality of evidence rather than as universally tested biological cut-offs. This distinction becomes particularly relevant at very low LDL-C concentrations and in lower-risk primary prevention, where relative treatment effects may remain favorable but the absolute benefit is smaller.

## 4. Pharmacological Therapy: From Statin Monotherapy to Individualized Combination Treatment

### 4.1. Statins and Treatment Reassessment

Statins remain the foundation of pharmacological LDL-C lowering in primary and secondary prevention. High-intensity treatment, or the maximally tolerated statin dose, is recommended when a reduction of at least 50% is required, whereas moderate-intensity therapy remains appropriate according to cardiovascular risk, treatment goals, age, comorbidities, and tolerability. The therapeutic strategy should not be framed as a choice between statin and non-statin treatment, but as selection of a regimen sufficiently potent to achieve both the recommended percentage reduction and absolute lipid goals [1]. Treatment response should be reassessed after initiation or modification of therapy. An inadequate reduction should prompt evaluation of adherence, statin dose, drug interactions, secondary dyslipidemia, and tolerability before pharmacological failure is assumed. However, repeated delays in intensification should be avoided when baseline LDL-C and cardiovascular risk make attainment of the goal with statin monotherapy unlikely.

### 4.2. Ezetimibe

Ezetimibe is generally the first non-statin agent added because it is orally administered, well tolerated, widely available, and lowers LDL-C by approximately 15–25% when combined with a statin. Fixed-dose statin–ezetimibe combinations can reduce pill burden and may facilitate long-term adherence, particularly when combination therapy is initiated early. IMPROVE-IT demonstrated that adding ezetimibe to simvastatin after acute coronary syndrome produced a modest but significant reduction in cardiovascular events, establishing clinical outcome benefit beyond statin monotherapy [15]. Ezetimibe is particularly suitable when a moderate additional LDL-C reduction is required. Its limited potency should nevertheless be anticipated: in patients who remain markedly above goal, ezetimibe alone may be insufficient and a more potent combination should be considered without unnecessary delay.

### 4.3. PCSK9 Monoclonal Antibodies

Evolocumab and alirocumab generally lower LDL-C by approximately 50–60% when added to background treatment. FOURIER and ODYSSEY OUTCOMES demonstrated reductions in cardiovascular events in patients with stable ASCVD and after acute coronary syndrome, respectively [6,16]. PCSK9 monoclonal antibodies are therefore particularly valuable in very-high-risk secondary prevention, familial hypercholesterolemia, severe primary hypercholesterolemia, and patients who remain substantially above goal despite maximally tolerated statin and ezetimibe. In addition to their marked LDL-C-lowering effect, these agents reduce Lp(a) concentrations by approximately 20–30%. However, they are not approved specifically for Lp(a) lowering, and the extent to which this effect contributes independently to cardiovascular benefit remains uncertain. Their main limitations concern cost, reimbursement, subcutaneous administration, and long-term persistence rather than uncertainty regarding cardiovascular efficacy [19].

### 4.4. Bempedoic Acid

Bempedoic acid provides an oral option for patients requiring additional LDL-C lowering, particularly when statin tolerance is limited. CLEAR Outcomes demonstrated a reduction in major cardiovascular events in patients unable or unwilling to take guideline-recommended statin doses, supporting its use as an evidence-based non-statin therapy [20]. Its LDL-C-lowering effect is less pronounced than that of PCSK9 monoclonal antibodies but is enhanced when combined with ezetimibe, including in a fixed-dose formulation. Treatment selection should consider the reduction required, preference for oral therapy, cost, and access to injectable agents. Hyperuricaemia, gout, cholelithiasis, and modest increases in hepatic enzymes or creatinine should be considered, particularly in predisposed patients [20].

### 4.5. Inclisiran

Inclisiran reduces hepatic PCSK9 synthesis and produces an LDL-C reduction of approximately 50%. After the initial dose and a second injection at three months, it is administered every six months by a healthcare professional. ORION-10 and ORION-11 demonstrated sustained lipid lowering in patients with ASCVD or an ASCVD risk equivalent whose LDL-C remained elevated despite background therapy [21]. Its infrequent administration may be advantageous in patients with poor persistence with daily medication or difficulty self-administering injections. However, inclisiran and PCSK9 monoclonal antibodies do not currently have an equivalent evidence base. Evolocumab and alirocumab have demonstrated cardiovascular outcome benefits in dedicated trials, whereas the pivotal inclisiran studies primarily evaluated LDL-C lowering and safety. Until dedicated outcome trials are completed, inclisiran should be considered a practical alternative for selected patients rather than a routine replacement for PCSK9 monoclonal antibodies [1,21].

### 4.6. Evinacumab and Homozygous Familial Hypercholesterolaemia

Evinacumab lowers LDL-C through inhibition of angiopoietin-like protein 3 and acts largely independently of residual LDL-receptor activity. In ELIPSE HoFH, it produced an approximately 49-percentage-point placebo-corrected reduction in LDL-C despite intensive background therapy [22]. Its role should therefore be restricted to specialist management of homozygous familial hypercholesterolaemia rather than general severe hypercholesterolaemia or hypertriglyceridemia. These patients frequently require multidrug treatment, including statins, ezetimibe, receptor-dependent PCSK9 therapy when effective, lomitapide, evinacumab, and lipoprotein apheresis.

### 4.7. Statin Intolerance

Symptoms occurring during statin therapy should not automatically lead to permanent withdrawal of the drug class. Evaluation should address the temporal relationship with treatment, interacting medications, secondary causes, and creatine kinase when clinically indicated. Rechallenge with a different statin, a lower dose, or an alternative schedule may identify a tolerable regimen [23]. Statin intolerance may be partial, when some exposure is tolerated but insufficient to reach the goal, or complete, when no clinically meaningful regimen can be maintained. Whenever possible, even a low tolerated dose should be combined with non-statin therapy rather than abandoned. In high-risk patients, investigation of symptoms and non-statin intensification may proceed in parallel to avoid prolonged exposure to markedly elevated LDL-C.

### 4.8. Moving Beyond a Rigid Stepwise Strategy

**Figure 2:**
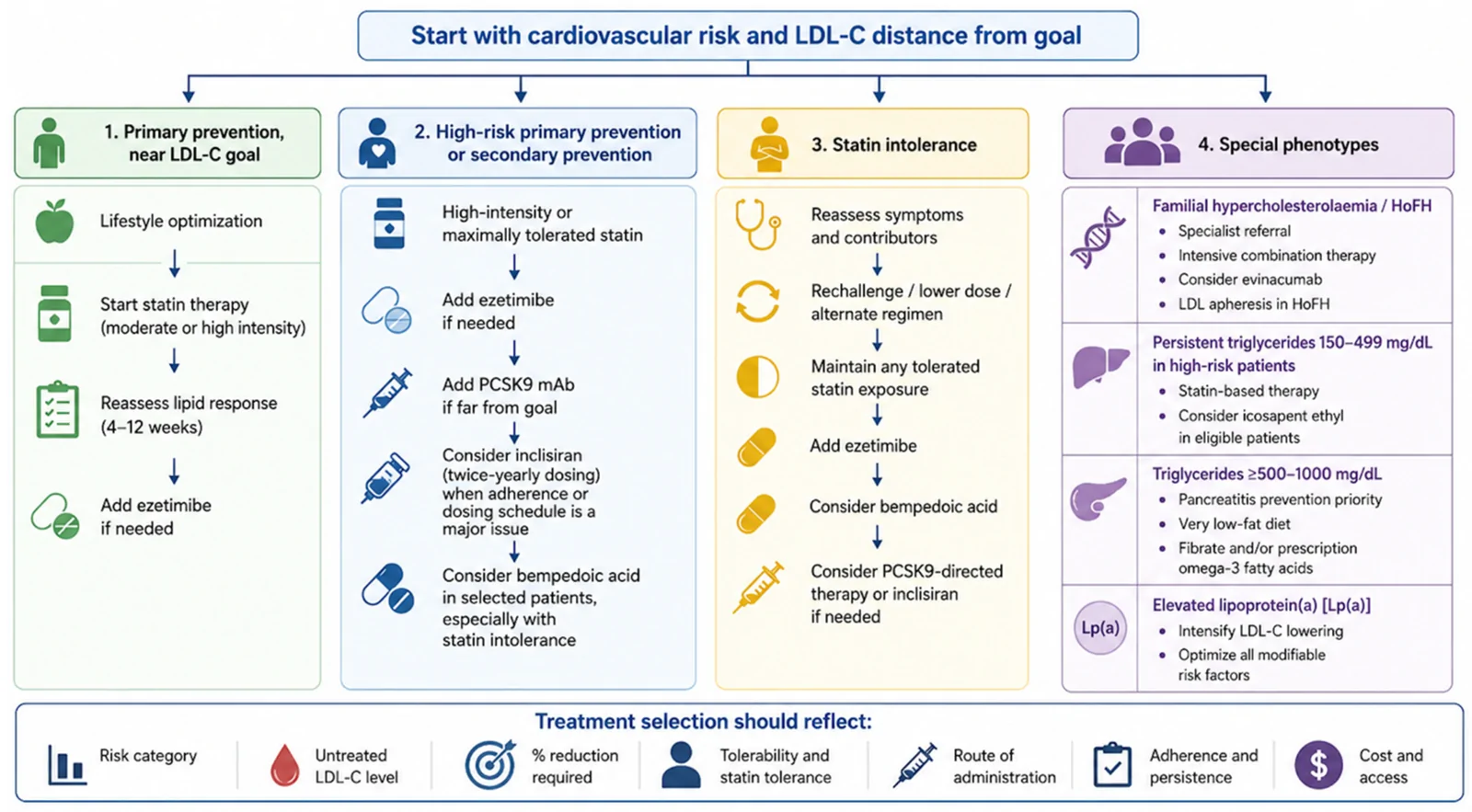
**Practical sequencing of lipid-lowering therapies according to cardiovascular risk, distance from the LDL-C goal, and clinical phenotype.** The choice and sequencing of lipid-lowering therapy should be guided by baseline cardiovascular risk, untreated LDL-C concentration, the magnitude of reduction required to achieve the recommended goal, and treatment tolerability. Statins remain the foundation of therapy, with ezetimibe, PCSK9 monoclonal antibodies, bempedoic acid, or inclisiran added according to the patient’s risk profile and distance from goal. A structured assessment and rechallenge strategy should be adopted in patients reporting statin-associated symptoms, while any tolerated statin dose should be maintained whenever possible. Specific clinical phenotypes, including familial hypercholesterolaemia, homozygous familial hypercholesterolaemia, persistent hypertriglyceridemia, and elevated lipoprotein(a), require tailored interventions and, when appropriate, specialist referral. Route of administration, adherence, persistence, cost, and access should also be incorporated into the final treatment decision. **Abbreviations:** HoFH, homozygous familial hypercholesterolaemia; LDL-C, low-density lipoprotein cholesterol; Lp(a), lipoprotein(a); mAb, monoclonal antibody; PCSK9, proprotein convertase subtilisin/kexin type 9.

The wider availability of lipid-lowering therapies makes a rigid stepwise algorithm increasingly difficult to justify when the LDL-C reduction required can be estimated from baseline. However, the evidence supporting early combination treatment is not equally strong for every therapeutic combination. Cardiovascular outcome benefit has been demonstrated for statins, ezetimibe, PCSK9 monoclonal antibodies, and bempedoic acid in defined populations, whereas inclisiran currently has robust LDL-C-lowering data but awaits definitive results from dedicated cardiovascular outcome trials. Moreover, most randomized trials have evaluated the addition of one agent to background therapy rather than immediate multidrug treatment versus conventional sequential escalation. Early combination therapy is therefore most compelling in very-high-risk patients or when monotherapy is predictably insufficient, while a sequential approach remains reasonable when the patient is close to goal or treatment tolerance is uncertain. The distinction between expected lipid-lowering efficacy and demonstrated cardiovascular outcome benefit remains essential when selecting among available therapies. The practical selection of lipid-lowering therapies according to risk profile, treatment goals, and clinical phenotype is summarized in Figure 2.

## 5. Hypertriglyceridemia, Triglyceride-Rich Lipoproteins, and Residual Cardiovascular Risk

The explicit inclusion of hypertriglyceridemia is one of the features that most clearly distinguishes the 2026 guideline from the previous US cholesterol recommendations. Elevated triglycerides frequently coexist with obesity, insulin resistance, type 2 diabetes, CKD, and mixed dyslipidemia and may indicate an increased burden of triglyceride-rich lipoproteins and cholesterol-enriched remnant particles. Because these particles contain ApoB, their contribution to cardiovascular risk cannot be assessed from the triglyceride concentration alone [1]. Management should distinguish two clinical objectives. In mild-to-moderate hypertriglyceridemia, the principal aim is reduction in ASCVD risk. When triglycerides are severely elevated, particularly at or above 1000 mg/dL, prevention of acute pancreatitis becomes an immediate priority. Evidence supporting a treatment for cardiovascular prevention should not automatically be extrapolated to pancreatitis prevention, or vice versa.

### 5.1. Persistent Mild-to-Moderate Hypertriglyceridemia

In adults with triglycerides between 150 and 499 mg/dL, management begins with correction of secondary causes, including poor glycaemic control, alcohol excess, obesity, hypothyroidism, CKD, refined-carbohydrate intake, and triglyceride-raising medications. Lifestyle intervention should address weight reduction when appropriate, physical activity, alcohol restriction, and improvement in dietary quality [1]. Statins remain the pharmacological foundation of ASCVD prevention. Although their direct triglyceride-lowering effect is moderate, they reduce ApoB-containing lipoproteins and have established cardiovascular outcome benefits. Treatment decisions should therefore be based on overall cardiovascular risk and achievement of LDL-C and non-HDL-C goals rather than on the triglyceride value in isolation. Selective ApoB measurement may be useful when residual particle burden is suspected despite apparently controlled LDL-C [1,14].

### 5.2. Icosapent Ethyl and Cardiovascular Risk Reduction

Icosapent ethyl may be considered in selected statin-treated adults with established ASCVD, or diabetes with additional risk factors, persistent triglycerides between 150 and 499 mg/dL, and LDL-C below 100 mg/dL [1]. In REDUCE-IT, icosapent ethyl, a highly purified ethyl ester of eicosapentaenoic acid, administered at 2 g twice daily, reduced the composite of cardiovascular death, nonfatal myocardial infarction, nonfatal stroke, coronary revascularization, or unstable angina by 25% relative to placebo. The absolute difference was 4.8 percentage points over a median of 4.9 years, corresponding to a number needed to treat of 21 [24]. The benefit was greater than would be expected from the modest reduction in triglycerides alone, suggesting possible additional biological effects. However, the findings should be attributed to the specific formulation and population studied rather than generalized to triglyceride lowering as a class effect. Hospitalization for atrial fibrillation or flutter occurred more frequently with icosapent ethyl, while serious bleeding was numerically increased. These risks should be considered particularly in patients with previous atrial arrhythmias or high bleeding risk [24]. The results of REDUCE-IT should not be extrapolated to non-prescription fish-oil supplements or other omega-3 formulations. In STRENGTH, a high-dose combination of eicosapentaenoic acid and docosahexaenoic acid did not reduce major cardiovascular events in statin-treated patients with atherogenic dyslipidemia. Given the discordant findings across omega-3 outcome trials, the evidence should be regarded as formulation- and population-specific rather than as a class effect. Icosapent ethyl may therefore be considered only in carefully selected statin-treated patients at high cardiovascular risk who resemble the population enrolled in REDUCE-IT [1,24,25].

### 5.3. Fibrates and the Limits of Triglyceride Lowering

Fibrates lower triglycerides through activation of peroxisome proliferator-activated receptor alpha, but routine addition to statin therapy has not demonstrated consistent cardiovascular benefit. In ACCORD Lipid, fenofibrate added to simvastatin did not significantly reduce the primary cardiovascular outcome in the overall population with type 2 diabetes. A possible benefit in participants with high triglycerides and low HDL-C arose from subgroup analysis and remains hypothesis-generating [26]. PROMINENT provided further evidence that lowering triglycerides does not necessarily reduce cardiovascular events. Pemafibrate lowered triglycerides, remnant cholesterol, and apolipoprotein C-III in patients with diabetes and atherogenic dyslipidemia but did not improve cardiovascular outcomes. ApoB increased slightly despite favorable changes in other lipid variables [27]. These findings support the interpretation that reducing triglyceride content without reducing the total number of ApoB-containing particles may be insufficient to lower ASCVD risk. They also reinforce the clinical value of non-HDL-C and ApoB when assessing residual risk in mixed dyslipidemia.

### 5.4. Severe Hypertriglyceridemia and Pancreatitis Prevention

When fasting triglycerides reach at least 500 mg/dL, and especially when they exceed 1000 mg/dL, management should also target pancreatitis prevention. Reversible contributors, including uncontrolled diabetes, alcohol intake, pregnancy, hypothyroidism, renal disease, obesity, and triglyceride-raising medications, should be addressed promptly [1]. Patients with very high triglycerides may require marked restriction of dietary fat, avoidance of alcohol and added sugars, and specialist dietary support. At levels of 1000 mg/dL or higher, a very-low-fat diet is generally required to reduce chylomicron production. Fenofibrate and prescription omega-3 fatty acids may be used as adjuncts when severe hypertriglyceridemia persists. Their purpose in this setting is triglyceride reduction and pancreatitis prevention rather than an assumed reduction in ASCVD events. Fenofibrate is generally preferred when combined with statin therapy; gemfibrozil should usually be avoided because it increases the risk of statin-associated myopathy [1]. Direct randomized evidence comparing different triglyceride targets for pancreatitis prevention remains limited. Recommendations are therefore based on the strong association between severe hypertriglyceridemia, chylomicronemia, and pancreatitis, together with the known lipid effects of available therapies.

### 5.5. Familial Chylomicronemia Syndrome and ApoC-III Inhibition

Persistent triglycerides above 1000 mg/dL despite correction of secondary causes should raise suspicion of a chylomicronemia syndrome. Familial chylomicronemia syndrome is a rare inherited disorder of the lipoprotein-lipase pathway characterized by persistent chylomicronemia, recurrent abdominal pain, and a high risk of pancreatitis. Conventional triglyceride-lowering agents may have limited efficacy because their effects often depend on preserved lipoprotein-lipase activity. The 2026 guideline recommends olezarsen, in addition to dietary treatment, for adults with familial chylomicronemia syndrome and fasting triglycerides of at least 1000 mg/dL. Olezarsen is an antisense oligonucleotide directed against apolipoprotein C-III messenger RNA [1,28]. In BALANCE, olezarsen substantially reduced triglycerides and was associated with fewer episodes of acute pancreatitis than placebo, although the trial included only 66 patients and relatively few events [28]. Its use should therefore remain confined to accurately diagnosed familial chylomicronemia syndrome within specialist care, with attention to long-term safety, cost, and access.

### 5.6. A Phenotype-Based Approach

Treatment of hypertriglyceridemia should be guided by the underlying phenotype and therapeutic objective. Mild-to-moderate elevations require correction of secondary causes, lifestyle intervention, and statin-based control of ApoB-containing lipoproteins. Icosapent ethyl may provide additional cardiovascular benefit in selected high-risk patients. Fibrates and prescription omega-3 therapies have a clearer role in severe hypertriglyceridemia when pancreatitis prevention is the priority, while ApoC-III inhibition represents a disease-specific treatment for familial chylomicronemia syndrome. The central principle is that neither every triglyceride elevation requires a triglyceride-specific drug nor every triglyceride-lowering agent reduces cardiovascular events. Clinical benefit depends on the lipoprotein phenotype, change in ApoB-containing particle burden, baseline risk, and the outcome for which the therapy has been shown to be effective. This is also an area in which a biomarker-centered interpretation may be misleading: the clinical significance of an elevated triglyceride concentration depends on the underlying phenotype and the therapeutic objective, rather than simply on the magnitude of triglyceride reduction achieved.

## 6. Special Populations: Tailoring Lipid Management Across the Life Course

The 2026 guideline consolidates recommendations for populations in whom treatment benefit and safety depend on age, comorbidities, reproductive status, competing risks, and cumulative atherogenic exposure.

### 6.1. Diabetes Mellitus

Adults aged 40–75 years with type 1 or type 2 diabetes should receive lipid-lowering therapy for primary prevention irrespective of baseline LDL-C. PREVENT-ASCVD is used mainly to refine treatment intensity rather than determine whether therapy should be initiated. A moderate-intensity statin is appropriate for many patients without ASCVD, while more intensive lowering is warranted when multiple risk factors, long diabetes duration, albuminuria, reduced kidney function, microvascular complications, smoking, hypertension, or high estimated risk are present. Patients with diabetes and established ASCVD should follow the secondary-prevention pathway [1]. CARDS supports this approach by showing that atorvastatin reduced first cardiovascular events in type 2 diabetes even without markedly elevated LDL-C [29]. Because diabetes often combines hypertriglyceridemia with numerous cholesterol-depleted ApoB-containing particles, non-HDL-C and selective ApoB measurement may help identify residual risk when LDL-C appears controlled [1,14]. In adults younger than 40 years or older than 75 years, treatment should be individualized according to disease duration, organ damage, frailty, life expectancy, and patient preferences.

### 6.2. Chronic Kidney Disease

Adults aged 40–75 years with stage 3 or 4 CKD should receive lipid-lowering therapy for primary prevention independently of baseline LDL-C or PREVENT-ASCVD risk. SHARP demonstrated that simvastatin plus ezetimibe reduced major atherosclerotic events in moderate-to-severe CKD, supporting treatment before dialysis [30]. The evidence is less favorable after maintenance dialysis has begun. In 4D and AURORA, atorvastatin and rosuvastatin lowered LDL-C but did not significantly reduce the primary cardiovascular outcomes [31,32]. Routine initiation of statin therapy solely for primary prevention in dialysis is therefore not supported to the same extent, although previously established therapy need not be discontinued automatically. Decisions should consider ASCVD history, treatment burden, life expectancy, and the high proportion of non-atherosclerotic cardiovascular events in advanced kidney disease.

### 6.3. Older Adults

Chronological age alone does not determine treatment benefit. Functional status, frailty, multimorbidity, cognition, polypharmacy, life expectancy, and individual priorities should guide decisions. PREVENT-ASCVD may be used up to 79 years of age. In adults aged 75 years or older with an estimated life expectancy of at least 2.5 years, a moderate-intensity statin may be reasonable when expected benefit outweighs treatment burden. Selective CAC assessment can assist when uncertainty remains, but should not replace evaluation of competing risks and time to benefit [1]. Meta-analysis supports statin efficacy in older adults, although evidence beyond 75 years is less robust in primary prevention [33]. In secondary prevention, age alone should not prompt de-intensification of effective therapy. Deprescribing may be appropriate in severe frailty or very limited life expectancy and should reflect overall goals of care rather than age or LDL-C alone.

### 6.4. Pregnancy, Pregnancy Planning, and Lactation

For most patients planning pregnancy without severe familial hypercholesterolaemia or established ASCVD, statins should be stopped one to two months before conception or when pregnancy is recognized and generally withheld during pregnancy and lactation [1]. In selected women with familial hypercholesterolaemia or clinical ASCVD, continuation may be considered after individualized assessment of maternal benefit and fetal risk. Observational data have not shown a clear independent increase in major congenital malformations, but they do not support routine treatment in women at low or moderate short-term risk [34]. Bile acid sequestrants may be considered because they are not systemically absorbed, while lipoprotein apheresis remains an option for homozygous familial hypercholesterolaemia or exceptionally severe LDL-C elevation with ASCVD. Most other lipid-lowering agents should generally be avoided during lactation because of insufficient safety data. The guideline also recognizes adverse pregnancy outcomes and reproductive conditions, including preeclampsia, gestational hypertension, gestational diabetes, preterm delivery, polycystic ovary syndrome, and premature menopause, as markers of future cardiovascular risk.

### 6.5. Children and Adolescents

The life-course strategy emphasizes early detection of inherited dyslipidemia. Universal lipid screening is recommended between 9 and 11 years of age, with repeat testing at 19 years and at least every five years thereafter. Earlier testing from two years of age is reasonable when familial hypercholesterolaemia, severe hypercholesterolaemia, or premature ASCVD is present in a close relative [1]. In children aged at least eight years with LDL-C ≥ 160 mg/dL and a phenotype consistent with familial hypercholesterolaemia, statin therapy is recommended when lifestyle measures have not produced an adequate response after three to six months. Long-term follow-up of treated children with familial hypercholesterolaemia supports slower progression of carotid atherosclerosis and fewer early-adult cardiovascular events [35]. Suspected homozygous familial hypercholesterolaemia requires urgent specialist referral because intensive combination therapy, evinacumab, or lipoprotein apheresis may be needed. These populations require treatment intensity and goals to be adapted to clinical context, safety, competing risks, and time to benefit. An important limitation is that the strength of evidence is not uniform across these populations. Recommendations for middle-aged adults with diabetes or non-dialysis CKD are supported by relatively robust cardiovascular outcome data, whereas decisions in adults older than 75–80 years without ASCVD, patients with severe frailty, pregnancy, dialysis, and children receiving newer non-statin therapies rely more heavily on subgroup analyses, observational evidence, or extrapolation. A common target-based framework therefore cannot be applied with the same degree of certainty across the life course. In these settings, time to benefit, competing risks, reproductive safety, treatment burden, and the availability of long-term safety data become as relevant as the achieved LDL-C concentration.

## 7. From Recommendations to Clinical Practice: Implementation, Adherence, Access, and Health Equity

The impact of the 2026 guideline will depend on its translation into routine care. Real-world studies show that many high- and very-high-risk patients remain untreated or do not undergo escalation despite LDL-C values above goal. In DA VINCI, only about one-third of treated European patients achieved the LDL-C goals later adopted by ESC/EAS. In SANTORINI, goal attainment rose from 20.1% to 30.9% after one year, while treatment remained unchanged in approximately two-thirds. In the US GOULD registry, therapy was intensified in only 17.1% of patients with ASCVD and suboptimal LDL-C during two years of follow-up [36,37,38,39]. These findings indicate that publication of more intensive recommendations does not automatically change practice. The main barriers include inaccurate risk classification, underestimation of the reduction required, delayed reassessment, concerns regarding adverse effects, poor adherence, administrative restrictions, and unequal access to specialist treatment.

### 7.1. Risk Recognition and Therapeutic Inertia

Risk underestimation can lead to selection of an inadequately potent regimen or acceptance of LDL-C values above the appropriate goal. In SANTORINI, physician-assigned risk frequently differed from central reassessment [36]. PREVENT-ASCVD, risk-enhancing factors, Lp(a), ApoB, and CAC may improve characterization, but additional precision is useful only when linked to a clear treatment pathway. Therapeutic inertia occurs when treatment is not initiated or intensified despite failure to achieve the recommended objective. A rigid stepwise approach may contribute to delay when each modification is followed by prolonged reassessment intervals. Estimating the required percentage reduction from baseline can support earlier combination therapy in very-high-risk patients or those markedly above target. This should be distinguished from appropriate individualization, such as temporary deferral because of pregnancy, severe frailty, treatment-related symptoms, diagnostic uncertainty, or informed patient preference.

### 7.2. Monitoring, Adherence, and Continuity of Care

A target-based strategy requires structured follow-up after treatment initiation or modification. Reassessment should consider both percentage reduction and achieved LDL-C and non-HDL-C concentrations. An inadequate response should prompt evaluation of adherence, prescribed dose, treatment interruptions, secondary causes, and interacting drugs before pharmacological failure is assumed. Responsibility for follow-up must be explicit, particularly after acute coronary syndrome. Discharge documentation should specify the lipid goal, treatment plan, timing of repeat testing, and the clinician responsible for acting on the result. Long-term adherence cannot be inferred from the prescription itself. Patients may never start therapy, take it intermittently, or discontinue it because of symptoms, cost, or an unclear understanding of benefit. Communication should relate lipid lowering to individual risk and address treatment burden, safety, and expected benefit transparently. Statin-associated symptoms should be evaluated without assuming complete intolerance; rechallenge and non-statin therapy may preserve effective LDL-C lowering. Fixed-dose combinations or less frequent administration may improve persistence in selected patients.

### 7.3. Cost, Access, and Health Equity

Cost and reimbursement can determine whether an appropriate prescription results in actual treatment. In an early US analysis, fewer than half of new PCSK9 inhibitor prescriptions received insurance approval and only 30.9% of patients ultimately obtained therapy; higher out-of-pocket costs were associated with greater abandonment [40]. Although access conditions have changed, the study illustrates how administrative procedures and affordability can interrupt care. Personalized lipid management may widen disparities when access to Lp(a), ApoB, CAC, specialist care, and newer therapies is uneven. In a nationally representative US analysis, statin use for primary prevention was lower among Black and Hispanic adults than among White adults within comparable risk categories. Insurance coverage and a usual source of care were associated with greater treatment use [41]. Race-free PREVENT equations avoid treating race as a biological variable, but they cannot eliminate inequities in screening, affordability, follow-up, or access. Similarly, inability to obtain CAC should not prevent treatment when a clear clinical indication already exists.

### 7.4. System-Level Strategies

Implementation requires simple system-level pathways combining automated risk assessment, clearly assigned follow-up, timely reassessment, and prompt intensification. Electronic alerts alone have shown limited benefit and should be specific, actionable, and integrated into workflow [42]. Advanced testing should be reserved for situations in which it can alter management, while effective prescribing, reassessment, and adherence support remain universally available.

## 8. ACC/AHA 2026 and ESC/EAS 2025: Convergence and Divergence in Contemporary Lipid Management

The 2026 ACC/AHA guideline and the 2025 ESC/EAS Focused Update share the same biological premise: cardiovascular risk is related to both the concentration and duration of exposure to ApoB-containing lipoproteins, and greater absolute LDL-C reductions provide greater benefit in patients at higher baseline risk. Both frameworks support early identification of dyslipidemia, intensive treatment of established ASCVD, explicit LDL-C goals, and addition of non-statin therapy when statins alone are insufficient. Their main similarities and differences are summarized in Table 1 [1,9]. The two documents are not structurally equivalent. ACC/AHA 2026 is a comprehensive replacement of the 2018 US guideline, whereas the 2025 ESC/EAS document is a focused update of selected parts of the 2019 European guideline. The 2025 update introduced or revised recommendations on SCORE2 and SCORE2-OP risk estimation, newer LDL-C-lowering therapies including bempedoic acid and evinacumab for homozygous familial hypercholesterolaemia, lipid-lowering treatment during the index hospitalization for acute coronary syndromes, Lp(a), pharmacological treatment of hypertriglyceridemia, statin therapy for primary prevention in people with HIV, statin therapy in patients at high or very high chemotherapy-related cardiovascular toxicity risk, and dietary supplements. Other core elements, including the LDL-C treatment goals and much of the general target-based framework, were retained from the 2019 ESC/EAS guideline rather than newly introduced in 2025 [9,43].

### 8.1. Risk Estimation and Classification

ACC/AHA recommends PREVENT-ASCVD in adults aged 30–79 years without known ASCVD. Its sex-specific, race-free equations integrate cardiovascular, renal, and metabolic variables and provide both 10-year and 30-year estimates [1,8]. ESC/EAS uses SCORE2 in apparently healthy adults younger than 70 years and SCORE2-OP between 70 and 89 years. These models estimate 10-year cardiovascular risk and are recalibrated across four European geographical regions [9,44,45,46]. The percentages generated by PREVENT and SCORE2 are not interchangeable because the models differ in populations, variables, age ranges, outcomes, and calibration. Multinational validation supports the performance of PREVENT beyond the United States, but its adoption should still consider local guidelines and treatment thresholds [10]. PREVENT gives greater operational weight to longer-term risk and may identify younger adults with low short-term risk but substantial anticipated exposure to elevated LDL-C. The European framework remains centered on 10-year risk but supplements quantitative estimation with predefined clinical categories and risk modifiers. ESC/EAS assigns several conditions directly to high- or very-high-risk categories, including documented ASCVD, selected diabetes phenotypes, CKD, familial hypercholesterolaemia, and markedly elevated individual risk factors. Very-high-risk status is therefore not restricted to secondary prevention. ACC/AHA uses this terminology mainly to identify secondary-prevention patients at greatest recurrent risk, although extreme subclinical coronary atherosclerosis may justify similarly intensive LDL-C lowering. Neither approach is intrinsically more preventive. The European model emphasizes regional calibration and direct clinical classification, whereas the US model gives a more structured role to PREVENT, risk-enhancing factors, and selective reclassification.

### 8.2. Lipid Goals, Imaging, and Biomarkers

The return of explicit LDL-C goals has produced substantial transatlantic convergence. Both frameworks generally support LDL-C below 70 mg/dL in high-risk patients and below 55 mg/dL in very-high-risk patients. The principal difference is therefore how patients are assigned to these categories rather than the final target itself. Both documents also retain the importance of percentage LDL-C reduction from baseline [1,9,43]. ACC/AHA gives non-HDL-C greater prominence through parallel treatment goals, while ESC/EAS retains LDL-C as the primary target and uses non-HDL-C and ApoB mainly as secondary measures in metabolically complex patients. CAC has a more structured role in the ACC/AHA pathway and is used after PREVENT and risk enhancers when the treatment decision remains uncertain. Increasing calcium burden supports more intensive treatment, while CAC ≥ 1000 may justify an LDL-C goal similar to that used in very-high-risk secondary prevention [1]. ESC/EAS also recognizes CAC as a risk modifier but additionally considers carotid or femoral plaque and other evidence of subclinical atherosclerosis [9]. Differences regarding Lp(a) and ApoB are now relatively limited. Both documents support at least one Lp(a) measurement during adulthood and more intensive control of modifiable risk factors when concentrations are markedly elevated. ApoB is recognized as useful when LDL-C underestimates atherogenic particle number, although ACC/AHA provides more explicit indications for selective testing. A potentially relevant difference concerns how Lp(a)-associated risk is communicated. The ACC/AHA guideline provides a more granular interpretation, linking progressively higher Lp(a) concentrations to approximate increments in relative ASCVD risk, from about 1.4-fold at 125 nmol/L to approximately two-fold at 250 nmol/L and four-fold at 430 nmol/L. The ESC/EAS update also recognizes a continuous relationship between Lp(a) and cardiovascular risk, but its clinical framework remains more strongly centered on the use of approximately 50 mg/dL (105 nmol/L) as a risk-modifying threshold. The US approach may therefore better convey that markedly elevated Lp(a) should carry substantially greater clinical weight than values just above the conventional threshold [1,9].

### 8.3. Pharmacological Implementation and Clinical Implications

Statins remain first-line therapy in both systems, followed by ezetimibe, PCSK9 monoclonal antibodies, bempedoic acid, or other agents according to the LDL-C reduction required, tolerability, evidence base, cost, and access. The distinction between a rigid European escalation strategy and an individualized American stepwise approach is misleading. Both documents support earlier combination treatment when statin monotherapy is predictably insufficient. ESC/EAS places particular emphasis on intensive treatment during hospitalization for acute coronary syndrome, whereas ACC/AHA emphasizes matching treatment potency to cardiovascular risk and distance from goal. The timing of treatment intensification is particularly relevant after acute coronary syndrome. The ESC/EAS framework recommends reassessment of LDL-C 4–6 weeks after treatment initiation or intensification, with further escalation when the goal has not been achieved. However, the 2025 Focused Update also moves beyond a purely sequential strategy by supporting intensive combination therapy during the index hospitalization when statin monotherapy is predictably insufficient. This approach aims to avoid leaving very-high-risk patients inadequately treated during the early post-ACS period [9]. Real-world data support the importance of this early treatment window. In the SWEDEHEART registry, greater LDL-C reduction between the index myocardial infarction and the 6–10-week follow-up visit was associated with lower subsequent cardiovascular risk [47]. More recent SWEDEHEART data similarly showed the lowest event rates among patients achieving early and sustained non-HDL-C control [48]. Although observational, these findings reinforce the concern that repeated stepwise escalation may unnecessarily prolong exposure to elevated atherogenic lipoproteins during a particularly vulnerable period after myocardial infarction. The two frameworks should therefore be viewed as different methods of translating similar evidence into distinct healthcare environments. PREVENT offers a broader long-term cardiovascular–kidney–metabolic assessment, while SCORE2 and SCORE2-OP provide regional calibration and extend formal estimation to older age. For clinicians, risk estimates and treatment thresholds should be interpreted within the system from which they were derived, while the selected regimen should be sufficiently potent to achieve and maintain the appropriate lipid goal.

### 8.4. Statins for Prevention of Cancer Therapy-Related Cardiotoxicity

The 2025 ESC/EAS Focused Update also endorses statin therapy in patients at high or very high risk of chemotherapy-related cardiovascular toxicity [9]. This recommendation deserves a cautious interpretation. STOP-CA showed a reduction in anthracycline-associated left ventricular dysfunction with atorvastatin in patients with lymphoma [49], whereas three smaller randomized studies produced less consistent findings [9]. Moreover, available trials have primarily evaluated changes in left ventricular function rather than major cardiovascular outcomes. The recommendation is therefore supported by a comparatively limited and heterogeneous evidence base relative to conventional indications for statin therapy in ASCVD prevention. Statins are attractive in this setting because of their established safety and potential cardioprotective effects, but the magnitude of benefit, optimal patient selection, and effect on long-term clinical outcomes remain uncertain [9,49].

## 9. Critical Appraisal, Unresolved Issues, and Future Directions

Several elements of the 2026 framework still rely on observational evidence or extrapolation. Future research should assess the clinical consequences of its risk thresholds, imaging pathways, biomarkers, and treatment algorithms.

### 9.1. From Predicted Risk to Expected Treatment Benefit

PREVENT-ASCVD improves contemporary risk estimation and adds a 30-year perspective that may be particularly relevant in younger adults. However, predicted risk and expected treatment benefit are not equivalent. Patients with the same calculated risk may obtain different absolute benefits according to age, untreated LDL-C, the magnitude and duration of the achievable reduction, competing risks, and adherence. The PREVENT thresholds used to support treatment decisions have not themselves been tested in randomized trials comparing treatment initiation with deferral. Future models should therefore estimate the benefit expected from a specific intervention, potentially through projected absolute risk reduction, number needed to treat, or gain in event-free life expectancy. This would be especially useful in younger asymptomatic adults, in whom short-term benefit may appear modest despite considerable cumulative exposure. Recent multinational validation supports the performance of PREVENT beyond the United States, but its application should continue to consider local calibration, guideline endorsement, outcome definitions, and treatment thresholds [10].

### 9.2. CAC-Guided Treatment

CAC provides powerful prognostic information and can refine treatment decisions when clinical assessment remains uncertain. Nevertheless, the ability to predict events does not prove that a CAC-guided strategy improves outcomes compared with treatment based on conventional risk assessment. Randomized or pragmatic trials should determine whether CAC-guided initiation or intensification improves adherence, goal attainment, cardiovascular outcomes, and cost-effectiveness. Relevant questions include the safety of temporary treatment deferral in selected patients with CAC zero, the optimal interval for reassessment, and the appropriate treatment intensity in those with extreme calcium burden. The LDL-C goal below 55 mg/dL proposed for CAC ≥ 1000 is supported mainly by observational event rates and extrapolation from secondary-prevention trials. Whether this specific target is superior to less intensive goals in asymptomatic patients remains uncertain. Radiation exposure, incidental findings, availability, and potential inequities should also be considered.

### 9.3. Lp(a)-Lowering Therapies and Inclisiran Outcomes

Universal adult Lp(a) measurement will identify many individuals with inherited cardiovascular risk, but current management remains centered on intensive control of LDL-C and other modifiable factors. RNA-based therapies can produce substantial reductions in Lp(a), although biochemical efficacy alone does not establish cardiovascular benefit [50,51,52]. As of August 2026, the major cardiovascular outcome programs remain ongoing. Lp(a)HORIZON is evaluating pelacarsen in patients with established cardiovascular disease and elevated Lp(a) (NCT04023552), OCEAN(a)-Outcomes is evaluating olpasiran in patients with ASCVD and elevated Lp(a) (NCT05581303), and ACCLAIM-Lp(a) is evaluating lepodisiran in adults with elevated Lp(a) and established or high cardiovascular risk (NCT06292013). Definitive cardiovascular outcome results are not yet available. Important unresolved questions include the magnitude of Lp(a) reduction required to translate into clinical benefit, the appropriate treatment threshold, the role of these therapies in primary prevention, and their potential interaction with subclinical atherosclerosis and background LDL-C lowering.

A similar evidence gap remains for inclisiran. Its pivotal trials demonstrated sustained LDL-C lowering but were not designed to establish definitive cardiovascular outcome benefit. ORION-4 (NCT03705234) and VICTORION-2 PREVENT (NCT05030428) remain ongoing cardiovascular outcome trials [53]. Until these studies report, the twice-yearly dosing schedule of inclisiran may offer practical advantages in selected patients, but it should not be considered to have the same outcome evidence as evolocumab or alirocumab.

### 9.4. Therapeutic Sequencing and Choice of Lipid Targets

The guideline permits flexibility in sequencing statins and non-statin therapies, but complete treatment pathways have rarely been compared directly. Most outcome trials evaluated the addition of one agent to background therapy rather than early combination treatment versus conventional stepwise escalation. Pragmatic trials should establish whether starting a sufficiently potent combination produces earlier and more sustained goal attainment. The optimal strategy is likely to vary: immediate combination therapy may be appropriate after acute coronary syndrome or when LDL-C is markedly above goal, whereas sequential treatment may remain reasonable in lower-risk patients requiring a modest additional reduction. Uncertainty also persists regarding the optimal lipid measure used to guide intensification. ApoB may better represent residual particle burden when discordant with LDL-C or non-HDL-C, particularly in diabetes, obesity, and hypertriglyceridemia. However, observational associations do not prove that an ApoB-guided strategy improves outcomes compared with conventional LDL-C-guided treatment [14].

### 9.5. Implementation and Equity

Increasingly sophisticated risk assessment may improve precision but also create opportunities for delay, loss to follow-up, and unequal access. PREVENT, CAC, Lp(a), ApoB, multiple treatment goals, and an expanding pharmacological armamentarium will have limited impact unless they are integrated into simple and reliable care pathways. Implementation studies should evaluate treatment initiation, time to reassessment, intensification, persistence, goal attainment, costs, and cardiovascular outcomes. Evidence also remains limited in adults older than 80 years without ASCVD, patients with severe frailty, pregnant patients with familial hypercholesterolaemia or clinical ASCVD, children receiving newer therapies, and individuals with advanced CKD. Greater representation by sex, ancestry, socioeconomic position, and geographical region is essential. Personalized prevention cannot be considered successful if advanced testing and effective therapies remain concentrated among patients with easier access to specialist care. Ultimately, individualized risk assessment must be linked to an understandable estimate of treatment benefit, a feasible therapeutic strategy, and equitable long-term access to care.

### 9.6. Different Levels of Evidence Behind Contemporary Recommendations

Recommendations presented within the same guideline may rest on substantially different levels of evidence. Intensive LDL-C lowering with statins, ezetimibe, and PCSK9 monoclonal antibodies is supported by randomized cardiovascular outcome trials, and bempedoic acid has demonstrated outcome benefit in a population with statin intolerance. By contrast, CAC-guided treatment thresholds are based predominantly on observational risk gradients and extrapolation, ApoB-guided treatment has not been tested against conventional LDL-C-guided management in outcome trials, and inclisiran currently has stronger evidence for lipid lowering than for cardiovascular event reduction. The same distinction applies to emerging Lp(a)-lowering therapies, for which large biochemical effects cannot yet be assumed to translate into fewer events. These differences in evidentiary certainty may be obscured when recommendations are translated into a single clinical algorithm. For clinicians, the relevant question is therefore not only what the guideline recommends, but also how directly the underlying evidence supports the proposed intervention in a particular patient. The principal strength of the 2026 framework is its more refined characterization of risk; its unresolved challenge is to demonstrate that greater precision in classification consistently translates into better clinical outcomes.

## 10. Conclusions

The 2026 ACC/AHA Multisociety Guideline represents a substantial evolution in US lipid management. By extending its scope beyond LDL-C to include non-HDL-C, ApoB, lipoprotein(a), triglyceride-rich lipoproteins, inherited lipid disorders, and severe hypertriglyceridemia, it better reflects the biological and clinical heterogeneity of atherogenic risk. PREVENT-ASCVD, explicit lipid goals, risk-enhancing factors, and selective use of CAC collectively move the US framework toward a more individualized approach. Personalization should not, however, imply indiscriminate use of biomarkers, imaging, or additional therapies. Each test should clarify risk or modify treatment, and every abnormal finding should be linked to an achievable therapeutic pathway. Comparison with the 2025 ESC/EAS Focused Update shows increasing transatlantic convergence, particularly for LDL-C goals below 70 mg/dL in high-risk patients and below 55 mg/dL in very-high-risk patients. The main differences lie in risk classification, regional calibration, and the operational use of imaging and biomarkers rather than in the biological principles underlying treatment. The expanding range of lipid-lowering therapies makes goal attainment increasingly feasible, but treatments with demonstrated cardiovascular outcome benefit should remain clearly distinguished from those supported mainly by lipid efficacy. More broadly, the recommendations are not supported by a uniform evidence base: some rest on randomized cardiovascular outcome data, whereas others remain pragmatic extensions of observational risk prediction, imaging findings, or surrogate lipid efficacy. Relevant uncertainties persist regarding CAC-guided strategies, specific Lp(a) lowering, inclisiran outcomes, ApoB-guided treatment, and the optimal timing of combination therapy. The principal challenge remains implementation. Greater precision will have limited impact without timely treatment initiation, structured reassessment, support for adherence, prompt intensification, and equitable access to diagnostic and pharmacological resources. Ultimately, the success of the guideline should be measured not by the number of new tests or therapies introduced, but by its ability to identify risk earlier, translate it into a clear treatment objective, and maintain effective lipid control over time.

## Figures and Tables

**Table 1:** Comparison of the 2026 ACC/AHA Multisociety Guideline and the 2025 ESC/EAS Focused Update for the management of dyslipidemia.

Domain	2026 ACC/AHA Multisociety Guideline	2025 ESC/EAS Focused Update
**Nature of the document**	Comprehensive replacement of the 2018 US guideline on blood cholesterol, with an expanded scope encompassing LDL-C, non-HDL-C, triglyceride-rich lipoproteins, Lp(a), ApoB, inherited disorders, and special populations.	Focused update of selected recommendations from the 2019 ESC/EAS guideline. Recommendations not specifically modified in 2025 remain based on the 2019 document.
**Primary-prevention risk model**	PREVENT-ASCVD replaces the Pooled Cohort Equations.	SCORE2 is recommended for apparently healthy adults younger than 70 years; SCORE2-OP is used from 70 to 89 years. SCORE2-Diabetes applies to selected patients with type 2 diabetes.
**Age range for quantitative risk assessment**	Adults aged 30–79 years without known cardiovascular disease.	Apparently healthy adults aged 40–89 years without known cardiovascular disease.
**Time horizon**	Estimates both 10-year and 30-year ASCVD risk, supporting greater attention to long-term exposure in younger adults.	Estimates 10-year fatal and nonfatal cardiovascular risk. The update recognizes the need for future lifetime-risk and treatment-benefit models.
**Model characteristics**	Sex-specific and race-free equations incorporating cardiovascular, renal, and metabolic variables. Optional models may include UACR, HbA1c, and the Social Deprivation Index.	Sex-specific models using age, smoking, systolic blood pressure, and non-HDL-C, recalibrated to four European geographical risk regions.
**Calculated risk categories**	Low: <3%; borderline: 3% to <5%; intermediate: 5% to <10%; high: ≥10% 10-year PREVENT-ASCVD risk.	Low: <2%; moderate: 2% to <10%; high: 10% to <20%; very high: ≥20% 10-year SCORE2/SCORE2-OP risk, together with direct classification based on specific clinical conditions.
**Approach to risk refinement**	Structured “Calculate, Personalize, and Reclassify” model: PREVENT is followed by consideration of risk-enhancing factors and selective CAC testing when uncertainty remains.	SCORE2/SCORE2-OP estimates are supplemented by clinical risk modifiers, biomarkers, social factors, and evidence of subclinical atherosclerosis, particularly near treatment thresholds.
**Conditions determining high or very-high risk independently of the score**	Diabetes, stage 3–4 CKD, and HIV infection between 40 and 75 years support LDL-lowering therapy independently of LDL-C. Very-high-risk terminology is used mainly to distinguish secondary-prevention patients with the greatest risk of recurrence.	Clinical ASCVD, unequivocal ASCVD on imaging, selected diabetes phenotypes, severe CKD, familial hypercholesterolaemia with ASCVD or another major risk factor, and markedly elevated individual risk factors can determine high or very-high risk without SCORE2 calculation.
**Coronary artery calcium**	CAC has a structured role in reclassification and may guide both LDL-C and non-HDL-C goals. Absolute score and age-, sex-, and race-standardized percentile are considered. CAC ≥ 1000 identifies an extreme-risk phenotype supporting an LDL-C goal < 55 mg/dL.	Increased CAC is a risk modifier in moderate-risk individuals or those near treatment-decision thresholds. CAC and coronary imaging are not recommended for population-wide screening, and specific CAC-based LDL-C goals are not systematically provided.
**Other imaging markers**	Risk reclassification is centered predominantly on CAC.	Carotid or femoral plaque burden and coronary imaging may also act as risk modifiers, providing a broader imaging-based assessment of subclinical atherosclerosis.
**Lp(a) measurement**	Measurement at least once during adulthood is recommended. Lp(a) ≥ 125 nmol/L or ≥50 mg/dL is a risk-enhancing factor; markedly elevated values carry progressively greater risk.	Measurement at least once during adulthood should be considered. Lp(a) > 50 mg/dL or ≥105 nmol/L may modify risk, especially in moderate-risk patients or those near treatment thresholds.
**Clinical response to elevated Lp(a)**	Supports more intensive LDL-C lowering and comprehensive control of all modifiable risk factors. Lp(a) is explicitly incorporated into the personalization stage of risk assessment.	Supports risk reclassification and more intensive management of LDL-C and other risk factors. The degree of elevation and the overall risk profile should be considered together.
**ApoB**	Selective measurement may identify residual lipoprotein-related risk after LDL-C and non-HDL-C goals have been reached, particularly with triglycerides > 200 mg/dL, diabetes, or achieved LDL-C < 70 mg/dL.	ApoB remains a secondary treatment measure, particularly in diabetes, obesity, metabolic syndrome, hypertriglyceridemia, or very low LDL-C, when LDL-C may underestimate particle burden.
**LDL-C goals in primary prevention**	<100 mg/dL for borderline or intermediate risk; <70 mg/dL for high risk. Percentage reduction remains an additional treatment objective.	Retained from the 2019 ESC/EAS guideline and not modified by the 2025 Focused Update: <116 mg/dL for low risk; <100 mg/dL for moderate risk; <70 mg/dL and ≥50% reduction for high risk; <55 mg/dL and ≥50% reduction for very-high-risk patients.
**LDL-C goals in secondary prevention**	<55 mg/dL for very-high-risk ASCVD; <70 mg/dL for the smaller group with ASCVD not meeting very-high-risk criteria.	Retained from the 2019 ESC/EAS guideline and not modified by the 2025 Focused Update: <55 mg/dL and ≥50% reduction for all very-high-risk patients, including clinical ASCVD. A goal < 40 mg/dL may be considered after a recurrent vascular event within two years despite maximally tolerated therapy.
**Non-HDL-C goals**	Explicitly restored as parallel treatment goals. The goal is generally 30 mg/dL above the corresponding LDL-C goal; <85 mg/dL is recommended in very-high-risk secondary prevention.	Retained from the 2019 framework: LDL-C remains the primary treatment target, while non-HDL-C and ApoB are secondary targets, particularly in atherogenic or mixed dyslipidemia.
**Pharmacological foundation**	Statins remain first-line treatment. Ezetimibe, PCSK9 monoclonal antibodies, bempedoic acid, inclisiran, and selected specialist therapies are added according to required LDL-C reduction, evidence base, tolerability, access, and patient preference.	Statins remain first-line treatment. Non-statin therapies with demonstrated cardiovascular benefit should be added when the goal is not achieved, with the choice based on the additional LDL-C reduction required, availability, cost, and patient preference.
**Combination treatment**	Supports individualized sequencing and earlier combination treatment when the baseline LDL-C and required reduction make statin monotherapy predictably insufficient.	Explicitly supports a “strike early and strong” approach in selected high-risk settings, particularly when early combination therapy is needed to reach the goal promptly.
**Acute coronary syndromes**	Managed through the very-high-risk secondary-prevention pathway, with an LDL-C goal generally below 55 mg/dL and use of sufficiently potent combination therapy.	Places particular emphasis on immediate intensive LDL-C lowering during the index hospitalization and early addition of therapies with proven cardiovascular benefit according to the reduction required.
**Inclisiran**	Recognized as an alternative option when its twice-yearly administration offers a practical adherence advantage; cardiovascular outcome evidence remains under investigation.	May represent an alternative to PCSK9 monoclonal antibodies, but the absence of completed cardiovascular outcome trials is explicitly acknowledged.
**Overall orientation**	More structured use of long-term risk, CAC, non-HDL-C goals, and individualized risk-enhancing factors within a contemporary cardiovascular–kidney–metabolic framework.	Greater reliance on geographically calibrated risk assessment, direct clinical risk categories, broader vascular imaging modifiers, and an established risk-based target system.

For the ESC/EAS framework, recommendations not specifically revised in the 2025 Focused Update remain derived from the 2019 ESC/EAS guideline. **Abbreviations**: ACC, American College of Cardiology; AHA, American Heart Association; ApoB, apolipoprotein B; ASCVD, atherosclerotic cardiovascular disease; CAC, coronary artery calcium; CKD, chronic kidney disease; EAS, European Atherosclerosis Society; ESC, European Society of Cardiology; HbA1c, glycated hemoglobin; HIV, human immunodeficiency virus; LDL-C, low-density lipoprotein cholesterol; Lp(a), lipoprotein(a); PREVENT, Predicting Risk of cardiovascular disease EVENTs; SCORE2, Systematic Coronary Risk Evaluation 2; SCORE2-OP, Systematic Coronary Risk Evaluation 2-Older Persons; UACR, urinary albumin-to-creatinine ratio. Data derived from references [1,8,9,43,44,45,46].

## Data Availability

No new data were created or analyzed in this study. Data sharing is not applicable to this article.
